# Genome-Wide Association Study Reveals That *PvGUX1_1* Is Associated with Pod Stringlessness in Snap Bean (*Phaseolus vulgaris* L.)

**DOI:** 10.3390/biology11040611

**Published:** 2022-04-18

**Authors:** Zhiyuan Liu, Shuo Gao, Helong Zhang, Zhaosheng Xu, Wei Qian

**Affiliations:** Institute of Vegetables and Flowers, Chinese Academy of Agricultural Sciences, Beijing 100081, China; liuzhiyuan01@caas.cn (Z.L.); shuo19119051@163.com (S.G.); zhanghelong@caas.cn (H.Z.); xuzhaosheng@caas.cn (Z.X.)

**Keywords:** snap bean, genome-wide association study, candidate gene, pod stringlessness, syntenic analysis

## Abstract

**Simple Summary:**

Using 138 snap bean accessions as plant materials, we investigated their suture strings across two years. With the goal of identifying the gene(s) responsible for the formation of suture strings, we conducted a genome-wide association study. A strong association signal was found in a 266.19 kb region on Chr02. Within the region, 23 candidate genes were identified. Importantly, the sequence and gene expression of *PvGUX1_1* differed significantly between sutured pods and non-sutured pods. In addition, *PvGUX1_1* was also a domesticated locus that diverged from *PvGUX1_2* during an early stage. The results obtained in this study can provide important information for the improvement of pod quality in snap beans.

**Abstract:**

Suture strings are a particularly important pod trait that determine the quality and texture of snap beans (*Phaseolus vulgaris* L.). The St locus on chromosome 2 has been described as a major locus associated with suture strings. However, the gene and genetic basis underlying this locus remain unknown. Here, we investigated the suture strings of 138 snap bean accessions across two years. A total of 3.66 million single-nucleotide polymorphisms (SNPs) were obtained by deep resequencing. Based on these SNPs, we identified a strong association signal on Chr02 and a promising candidate gene, *PvGUX1_1.* Further analysis revealed that the 2 bp deletion in the exon of *PvGUX1_1* was significantly associated with stringlessness. Comparative mapping indicated that *PvGUX1_1* was a domesticated locus and diverged from *PvGUX1_2* during an early stage. Our study provides important insights into the genetic mechanism of suture string formation and useful information for snap bean improvement.

## 1. Introduction

Snap bean (*Phaseolus vulgaris* L.) is a type of common bean that is harvested before the seeds mature and eaten as a vegetable. Immature snap bean pods are succulent and rich in protein, carbohydrates, vitamin C, vitamin K, and carotenoids [1]. Therefore, the whole pods of snap bean are used for cooking or are preserved for freezing and canning [2]. The snap bean is mainly consumed in North America, Europe, the Middle East, Africa, and Asia. In recent years, China has become the largest producer of snap beans globally [3].

Improving pod quality is a major objective for snap bean breeding [4]. Some pod characteristics, including pod length, pod shape, spur length, and the absence or presence of suture strings, have made the snap bean more palatable than the dry bean (another type of common bean in which the mature seed is consumed) [5]. A snap bean with a straight, smooth pod that lacks suture strings is preferred in the fresh market. The fiber string along the suture is usually discarded before being eaten. Thus, the absence of suture strings is more popular with consumers and facilitates the commercial processing of snap beans.

Reducing suture strings in snap beans is crucial, and the key to this effort lies in understanding the genetic basis of the formation and development of suture strings. The inheritance analysis of suture strings revealed that stringlessness was governed by a dominant gene, *St*, in the common bean [6]. Quantitative trait locus (QTL) analysis located the *St* gene on chromosome Pv02 in the common bean [7]. Working with a recombinant inbred line derived from the dry bean and snap bean, a strong QTL, PST2.2, was also found on Pv02, accounting for 32% of total genetic variation in a recombinant inbred line [2]. As the reduction of suture strings and pod wall fibers commonly leads to pod indehiscence in the common bean, the indehiscent gene *PvIND* (a homolog of the Arabidopsis INDEHISCENT gene, *IND*) mapped near the St locus was predicted to be the St gene. However, there was incomplete co-segregation of *PvIND* and the St locus and a lack of polymorphisms with dehiscent/indehiscent phenotypes, suggesting that *PvIND* was not the *St* gene [8]. Recently, a single QTL, qPD5.1-PV, determining pod indehiscence was identified on chromosome Pv05 [9]. In the attempt to identify the candidate gene underlying the QTL, a BC4/F4 introgression line population was used to narrow down the QTLs in a 22.5-kb region and *PvMYB26* was identified as the best candidate gene based on mapping and gene expression patterns [10]. In addition, several genes or QTLs were also found to be associated with pod dehiscence, such as *PvPdh1* on chromosome Pv03 and QTLs on Pv08, Pv05, and Pv09 [11].

The first common bean reference genome was published in 2014 [12], which made it possible to use different strategies to identify candidate genes and molecular markers for important agronomic traits. The genome-wide association study (GWAS) is an approach based on using the numbers of single-nucleotide polymorphisms (SNPs) to test the association of desired traits. Due to the reduced cost of resequencing and the repeatability of SNPs in the genome, GWAS has been performed using various landraces and breeding lines in the common bean. These studies have focused on grain yield [13,14,15], flowering time [16], resistance to disease [17], resistance to pod shattering [11], grain mineral content [18], drought resistance [19], and abiotic stress [20]. However, few studies have focused on specific traits in the snap bean [21]. Pod stringlessness is particularly crucial in snap beans. Therefore, the objective of this study was to identify the candidate gene associated with this trait as a basis for further improving the quality of the snap bean.

## 2. Materials and Methods

### 2.1. Plant Material and Resequencing

One hundred and thirty-eight snap bean accessions obtained from the Institute of Vegetables and Flowers at the Chinese Academy of Agriculture Sciences (CAAS), including landraces, elite lines, and breeding lines, were grown between March and June in 2019 and 2020 (Supplementary Table S1). These seeds were grown in mixed nutrient soil at a greenhouse in Beijing (40° N, 116° E). The plants were watered using automatic drip irrigation every 2–3 days throughout entire growth period. The field away from plant was covered with a mulching plastic sheet to reduce the weeds.

Young leaves at the unifoliate growth stage were collected from each accession were collected, flash-frozen in liquid nitrogen, and stored in an ultra-low-temperature freezer. Genomic DNA was isolated for each genotype using the Plant Genomic DNA kit (Tiangen, Beijing, China) following the manufacturer’s instructions. The integrity of the gDNA was determined on 1% agarose gels. The concentration and quality of the gDNA were measured using a NanoDrop2000 Spectrophotometer (Thermo Fisher Scientific, Wilmington, DE, USA). The DNA library was constructed according to the manufacturer’s instructions for the TruSeq nano DNA kit (Illumina). The libraries were genotyped using an Illumina HiSeq 2000 (125PE) sequencer at the facilities of Berry Genomics Co. Ltd., Beijing, China, as previously described [15].

### 2.2. Measurement of Pod Sutures

At the green mature stage, 10 fresh pods from different plants of each accession were randomly chosen to measure the pod suture strings. The 10 pods from 10 individuals served as technical replications. The strings were evaluated for both qualitative traits and quantitative traits. For the qualitative traits, the pod strings were scaled 0–1 (0 = no suture strings, 1 = presence of suture strings). For the quantitative traits, the ratio (string length/pod length) of each pod was measured. The average ratio value of 10 pods and the scale rating of pods were both used for the GWAS analysis.

### 2.3. Expression Analysis of PvGUX1_1

Three stages (T1 for pod elongation, T2 for pod development, and T3 for pod maturity) of R02 (non-sutured) pods and R05 (sutured) pods were sampled. The total RNA of the three stages of the pods in sutured pods and non-sutured pods was extracted and converted to cDNA using a Reverse Transcription Kit (TransGen Biotech, Beijing, China) according to the manufacturer’s instructions. Quantitative real-time PCR was performed with SYBR Green (Vazyme Biotech, Nanjing, China), and the data collection was performed on the QuantStudioTM 6 Flex system (ABI, Life, Carlsbad, CA, USA) according to the manufacturer’s instructions. The primers were synthesized by Sangon Biotech (Shanghai, China). The relative expression levels of *PvGUX1_1* were compared with those of β-actin, and the expression fold changes were calculated using the 2^−^^△△^^Ct^ method. Each qRT-PCR reaction was performed in triplicate. The sequences of the primers used for qRT-PCR in this study are shown in Supplementary Table S2.

### 2.4. Variant Calling and Annotation

The raw paired-end reads were initially filtered by fastp (v0.20.0) software with the following parameters: -q 30. Next, the clean reads were aligned with the common bean reference genome V2.1 [10] using the MEM algorithm of BWA (v0.7.17-r1188) [22].

The SortSam and MarkDuplicates tools in PICARD (v1.127) were used to sort the mapping results and mark the duplicate reads (https://broadinstitute.github.io/picard/, accessed on 22 September 2020). In addition, realignment around InDels was conducted by RealignerTargetCreator and IndelRealigner in GATK (v3.2) [23]. The variant was called by the UnifiedGenotyper module in GATK and SAMTOOLS (v1.6-3-g200708f) [22]. The two variant results were combined and further filtered to obtain a credible variant dataset using the GATK SelectVariants and VariantFiltration subcomponents. The credible variant dataset was employed to recalibrate and realign results using the BaseRecalibrator and PrintReads of GATK. The SNP and InDel were again called against the recalibrated results. Finally, a vcf file including all the samples and variants was generated and further filtered using vcftools software (0.1.15) with the following parameters: -max-missing 0.95 -maf 0.05 -min-alleles 2 -max-alleles 2 -recode -recode-INFO-all [24].

The functional annotation of variants was performed using ANNOVAR software (Version:2017-07-17) [25].

### 2.5. Population Genetics Analyses

To analyze the population structure, the reduced SNPs were employed based on the value of the correlation coefficient (r^2^), where SNPs with a strong linkage disequilibrium (LD) (r^2^ > 0.2) within a 50 kb window were discarded using plink (v1.90b6) software with the following parameters: -indep-pairwise 50 10 0.2 [26]. To estimate the optimal sub-population, a cross-validation procedure was conducted with ADMIXTURE (v1.3.0) runs from K = 2 to 16 [27]. A neighbor-joining tree of 138 snap bean accessions was constructed using Phylip 3.68 [28] software based on a distance matrix. The bar plots of sub-populations and the phylogenetic tree were plotted using the itol website (https://itol.embl.de/, accessed on 5 October 2020).

### 2.6. Linkage Disequilibrium Analysis

The correlation coefficient (r^2^) of pairwise SNPs within a 1000 kb window from all chromosomes was used to estimate LD decay, which was calculated and plotted using PopLDdecay software [29]. LDBlockShow software was used to calculate and display LD blocks in candidate regions (https://github.com/BGI-shenzhen/LDBlockShow, accessed on 15 October 2020).

### 2.7. Genome-Wide Association Analysis

High-quality SNPs were used for the GWAS analysis in the R package GAPIT [30]. To reduce false positives and improve statistical power, the ‘Q + K’ approach was employed. The kinship matrix (K) was calculated with the default method in GAPIT. The significant threshold (−log_10_*P*) was Bonferroni-corrected as −log_10_*P* = 7.86. The Manhattan plot was run by the CMplot package in R 3.6.1 (https://github.com/YinLiLin/CMplot, accessed 29 October 2020).

### 2.8. Analyses of Collinearity and Synteny

The genome sequence information of the common bean (*Phaseolus vulgaris* V2.1) and cowpea (*Vigna angularis* V1.2) was downloaded from phytozome 13. The genomes of soybean (*Glycine max* 109) (www.plantgdb.org/XGDB/phplib/download.php?GDB=Gm, accessed on 1 November 2020) and pea (*Pisum sativum*) (https://urgi.versailles.inra.fr/Species/Pisum, accessed on 1 November 2020) were downloaded from public databases. The analysis of collinearity and synteny between the four legumes was implemented with MCScan (Python version) (https://github.com/tanghaibao/jcvi/wiki/MCscan-[Python-version], accessed on 25 November 2019). Proteins with a similarity of over 90% on *PvGUX1_1* in the common bean, soybean, cowpea, and pea were identified using BLASTP with an e value < 10^−5^. The neighbor-joining tree from the orthologue gene of *PvGUX1_1* was constructed using MEGA X [31] with default parameters.

## 3. Results

### 3.1. Pod Suture String Phenotyping

**Figure 1:**
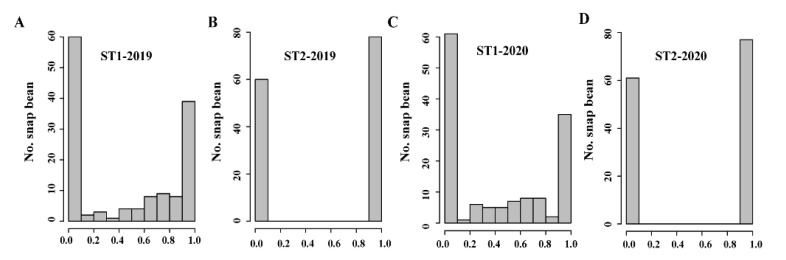
Histograms of pod suture strings in 138 snap bean accessions. (**A**) The ratio (string length/pod length) was measured in 2019. (**B**) The rating (1 for the presence of a string and 0 for the absence of a string) was determined in 2019. (**C**) The ratio was measured in 2020. (**D**) The rating was determined in 2020. ST1: the ratio of string length to pod length; ST2: the rating of whether there are strings (1 for the presence of a string, and 0 for the absence of a string).

The pod suture strings of 138 snap bean accessions were investigated based on the ratings and ratios (Figure 1). For the rating, the presence of strings was defined as 1; the absence of strings was defined as 0. The ratings were investigated in 2019 (Figure 1B) and 2020 (Figure 1D). A total of 60 accessions were stringless, whereas 78 accessions had suture strings in 2019 (ST2-2019) (Figure 1B). However, five accessions showed different ratings in 2020 (ST2-2020). For the ratios, the average ratio values (string length/pod length) of 10 pods in each accession were measured in 2019 (ST1-2019) and 2020 (ST1-2020) (Figure 1A,C). The analysis of correlation for the ratios showed that there was a significantly high correlation of 0.93 (*p* = 0.00015) between 2019 and 2020.

### 3.2. Resequencing of Snap Bean Accessions

**Figure 2:**
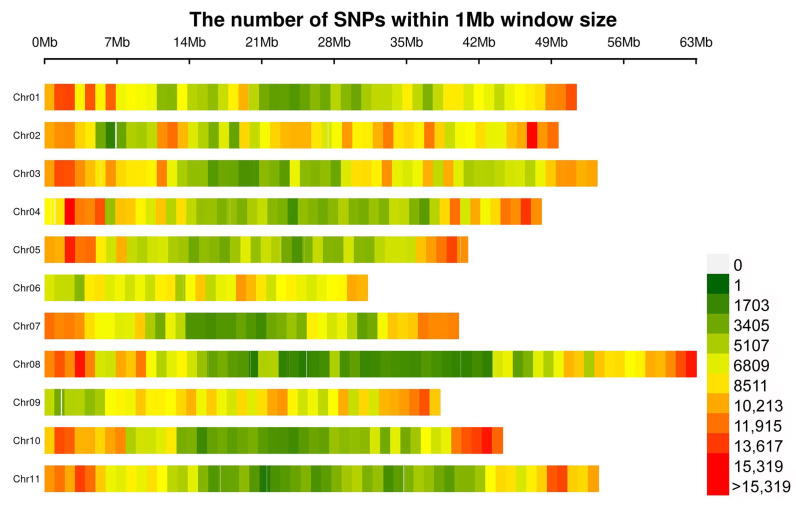
The number of SNPs within a 1 Mb window size across common bean chromosomes.

The whole-genome resequencing of 138 accessions produced a total of 3.08 billion raw paired-end reads and 0.92 Tb bases, which had an approximately 11.4-fold sequence depth, ranging from 10.2- to 13.5-fold. After being filtered, 2.71 billion clean reads were retained. Mapping against the common bean genome V2.1 resulted in 5,130,030 SNPs and 1,524,528 InDels. Further filtering (bi-allelic, missing data < 0.05, minor allele frequency > 0.05) identified 3,656,683 high-confidence SNPs and 626,853 InDels. Among these variants, 3,589,978 SNPs and 618,666 InDels were placed on chromosomes; 66,705 SNPs and 8187 InDels were on scaffolds. The distribution of these SNPs across the genome was uneven (Figure 2). Most SNPs were located in Chr02 (411,294), and the fewest SNPs were found in Chr06 (238,452). In addition, the frequency of SNPs in Chr02 (8.28 SNPs/kb) was the highest, while the frequency of SNPs in Chr08 (5.97 SNPs/kb) was the lowest (Supplementary Table S3).

To investigate the distribution regions of these variants across the genome, we carried out variant annotation and found that 146,882, 512,153, 279,102, and 244,805 SNPs and 4180, 53,742, 30,812, and 28,930 InDels were located in exons, introns, upstream, and downstream, respectively. Furthermore, of these SNPs in exons, 65,001 nonsynonymous, 718 stopgain, and 171 stoploss SNPs were annotated, which resulted in amino acid changes, premature stop codons, or longer transcripts. Similarly, of these InDels in exons, 697 frameshift insertion, 1091 frameshift deletion, three stoploss, and 49 stopgain InDels were annotated, which also influenced protein sequences.

### 3.3. Population Structure and LD Analysis

**Figure 3:**
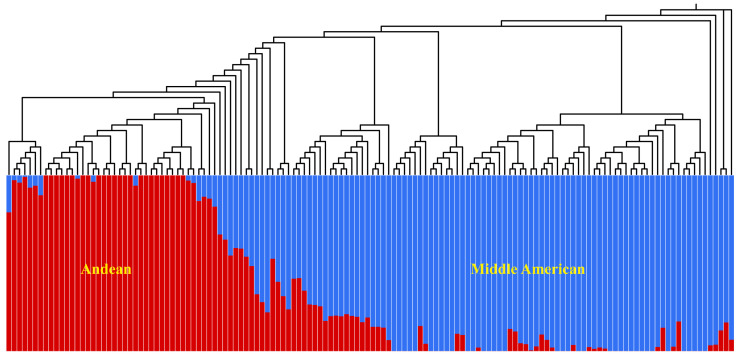
Neighbor-joining tree and population structure analysis using 97,841 single-nucleotide polymorphisms (SNPs). The genepools are colored with red for Andean and blue for Middle American ancestry.

The analysis of population structure allows researchers to understand the genetic relationships between and origins of species. After removing the SNPs with a strong LD (r^2^ ≥ 0.2), 97,841 SNPs were generated and used to implement a population structure analysis with Admixture. The use of K = 2 divided the 138 genotypes into two genetic groups, which was in agreement with two domesticated genepools (Andean and Middle American) (Figure 3). Among the 138 genotypes, 40 genotypes had predominantly Andean ancestry, and 30 genotypes contained a level of hybridization, suggesting that a high degree of intercrossing between the genepools occurred within snap beans.

We further analyzed the LD decay across the genome (Supplementary Figure S1). The average LD decay of the whole genome was 631.4 kb (r^2^ dropped to half of its maximum value), which was faster than that of common bean (107 kb) [15], cultivated soybean (150 kb) [32], and cultivated rice (123 kb for indica and 167 kb for japonica) [33]. In addition, we found that the rate of LD decay in different chromosomes varied from 184 kb (Chr10) to 976 kb (Chr01) (Supplementary Table S4).

### 3.4. Genome-Wide Association Study for Pod Stringlessness

**Figure 4:**
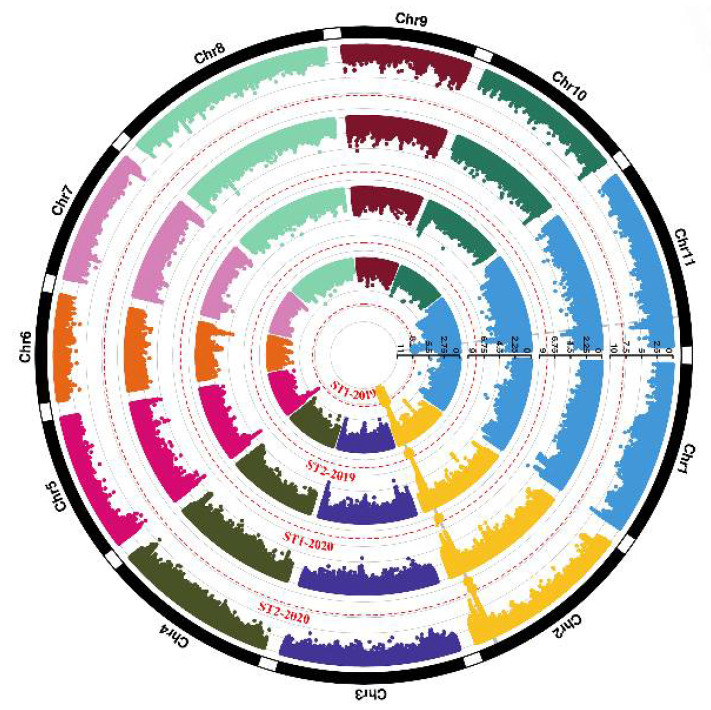
Circular Manhattan plots of a genome-wide association study (GWAS) for pod stringlessness. The inner circle to the outer circle represents ST1-2019, ST2-2019, ST1-2020, and ST2-2020, respectively. The red dashed lines of each circle indicate the threshold (7.86). Single-nucleotide polymorphisms (SNPs) over the threshold are highlighted.

To find out genetic loci controlling pod stringlessness, we implemented GWAS for four traits (ST1-2019, ST2-2019, ST1-2020, and ST2-2020) using 3,656,683 SNPs (Figure 4). The Q2 and relatedness kinship matrix as covariates were taken into account to reduce false positives in the GWAS analysis with a compressed mixed linear model. The −log_10_(*P*) = 7.86 was set as a genome-wide significance threshold based on Bonferroni correction. Strong association signals were used to identify candidate regions and screen candidate genes.

A total of 205 loci were identified with −log_10_(*P*) > 7.86 for ST1-2019. Of 205 SNPs, 204 were located at Chr02, and 1 was located at Chr11 (Supplementary Table S5). The peak signal was located at Chr02:44026689 (−log_10_(*P*) = 10.08), accounting for 14.53% of phenotypic variation. The major locus Chr02:44248269 (−log_10_(*P*) = 8.60) was significantly associated with ST2-2019. Furthermore, strong signals were both found at Chr02:44194640 for ST1-2020 (−log_10_(*P*) = 8.49) and ST2-2020 (−log_10_(*P*) = 9.62). Taken together, the peak SNPs for the four traits were all located in adjacent physical regions in chromosome 2, which suggested that pod stringlessness was under the control of a major locus.

### 3.5. Identification of Candidate Genes for Pod Stringlessness

**Figure 5:**
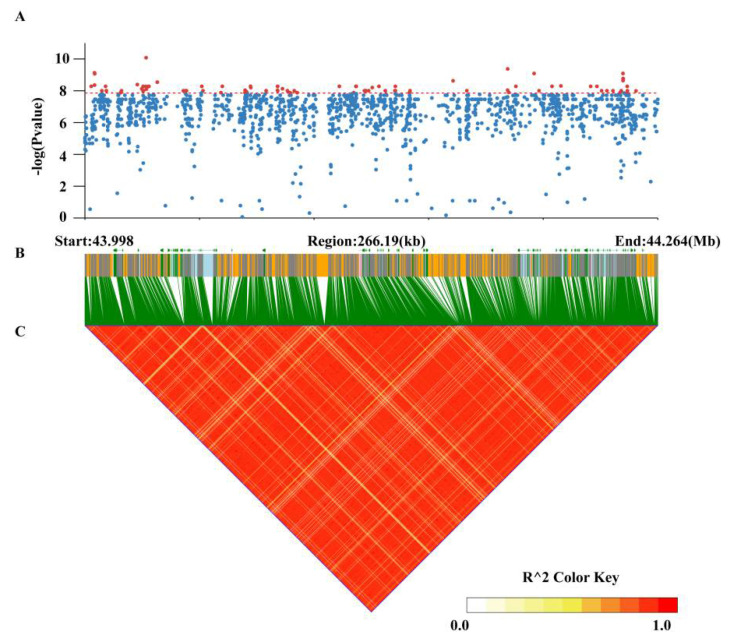
Manhattan plots and linkage disequilibrium (LD) heatmap over 266.29 kb around significant single-nucleotide polymorphisms (SNPs) on chromosome 2. (**A**) Manhattan plots of ST1-2019. The red dashed line represents the threshold (7.86). SNPs over the threshold are highlighted in red. (**B**) Annotated genes in the region. These CDS, introns, UTR, and intergenic regions are shown in green, light blue, pink, and orange, respectively. (**C**) The LD heatmap over the region. Colors are coded according to the r^2^ color key.

To identify the candidate regions associated with the significant SNPs, we carried out haplotype analysis in the whole genome. We found that these peak SNPs for the four traits all resided on the same linkage disequilibrium (LD) block (Chr02:43998258–44264446) (Figure 5). These genes located in the block were likely responsible for the formation of stringlessness. Table 1 shows these genes and their homologous genes in Arabidopsis. A total of 23 putative genes were annotated in this block based on the common bean reference genome V2.1. A total of 18 out of 23 genes were functionally annotated, and 15 genes had homologous genes in Arabidopsis. Furthermore, 102 SNPs, including 43 nonsynonymous and 59 synonymous SNPs, and 6 InDels, including two frameshift deletions distributed in the coding areas of these genes, were also identified (Table 2).

**Figure 6:**
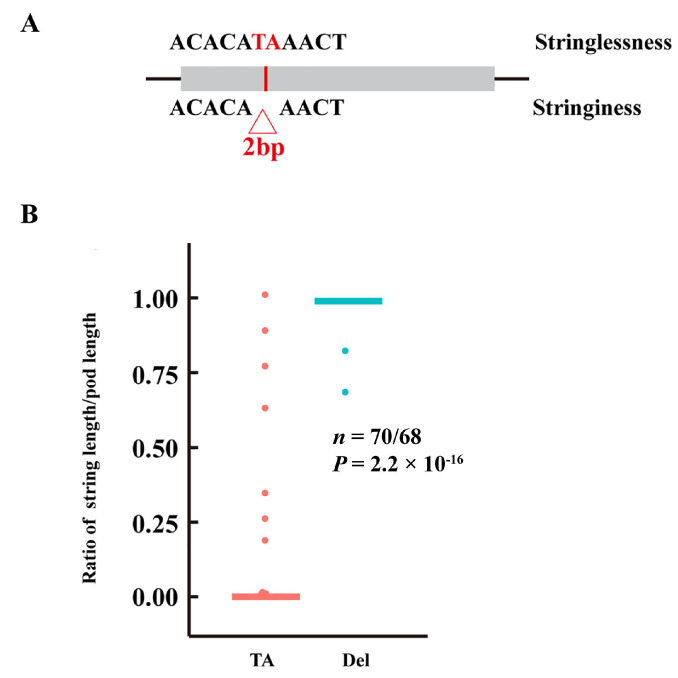
The identification of a 2 bp deletion in Phvul.002G270800. (**A**) Structure of Phvul.002G270800. The red base represents the 2 bp deletion in Phvul.002G270800. (**B**) Box plot of the ratio of string length/pod length for the 2 bp deletion in Phvul.002G270800.

A 2 bp deletion in the exon region was identified in *Phvul.002G270800*, an ortholog to *AtGUX1*, which is responsible for secondary wall deposition in Arabidopsis. The 2 bp deletion introduced a premature stop codon that truncated the protein to 64 amino acids. To verify the deletion, we cloned the gene from two suture and non-suture accessions (Supplementary Figure S2). The result was similar to the finding obtained by resequencing. Additionally, the deletion of 2 bp was significantly correlated with pod stringlessness (2.2 × 10^−16^) (Figure 6). We identified another a 2 bp deletion in gene *Phvul.002G271600*; the 2 bp deletion led to a premature stop codon and truncated the protein with only 22 amino acids. However, the InDel was weakly associated with pod stringlessness (2.23 × 10^−6^) and the function of this gene was unclear, suggesting that Phvul.002G276100 maybe not be the key gene.

The most abundant nonsynonymous SNPs were found in *Phvul.002G271700. Phvul.002G271700,* encoding a NAC domain protein, carried eight nonsynonymous SNPs. Among these SNPs, K120I was significantly associated with pod stringlessness (1.39 × 10^−8^), whereas other SNPs exhibited a weak association.

Three nonsynonymous SNPs, T32C, C604T and C737T, were identified in *PvIND* (*Phvul.002G271000*). The SNPs T32C and C604 showed a weak association (*p* = 6.42 × 10^−8^ and 6.79 × 10^−6^) with pod stringlessness, while C737T showed no association (*p* = 0.24).

### 3.6. Syntenic Analysis of the Candidate Region of the Common Bean and Other Legumes

**Figure 7:**
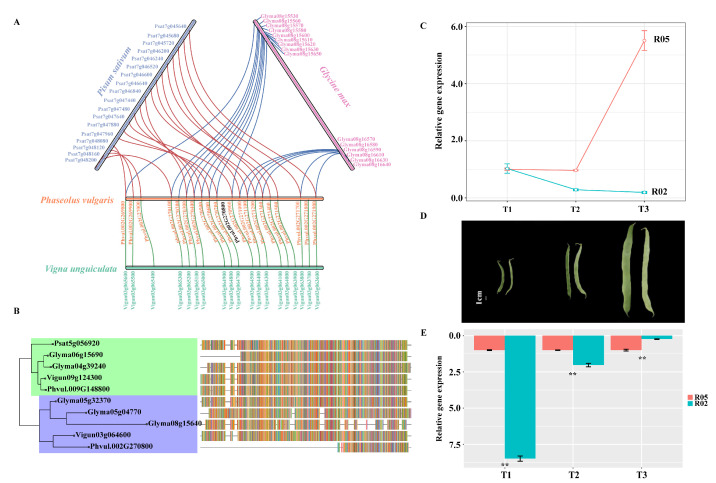
The analysis of phylogeny and expression of *PvGUX1*. (**A**). Syntenic analysis of the candidate region between common bean and other legumes. (**B**). Phylogenetic tree for *PvGUX1*. Colors located at the right side of each sequence represent their amino acid composition. (**C**). Gene expression of *PvGUX1_1* compared among three pod development stages for one accession. (**D**). Morphology of stringless and stringed pod development stages T1–T3. (**E**). The gene expression of *PvGUX1_1* compared between stringless and stringed accessions for different pod development stages. *p* values were calculated using Student’s t test (** *p* < 0.01).

To identify the function and relation of the candidate gene, we performed a syntenic analysis within the candidate region of the common bean against three legumes, including soybean (*Glycine max*), cowpea (*Vigna angularis*), and pea (*Pisum sativum*). The common bean, cowpea, and soybean are members of the Phaseoleae tribe, whereas pea belongs to the Fabeae tribe [34]. Amongst these legumes, the majority of cowpeas are stringless, while the common bean and pea have stringless and stringed types. In the Phaseoleae tribe, the common bean and cowpea are the two most closely related crop species of the four legumes analyzed. They also exhibited a high degree of synteny (Figure 7A), in which 19 of 23 genes were orthologous. Although large-scale synteny with soybean was also observed, the homologous genes in soybean were divided into two regions (Glyma08g15530–Glyma08g15650 and Glyma08g16570–Glyma08g16640) on chromosome 8. In contrast, the pea chromosome exhibited a large rearrangement with the common bean.

Overall, these candidate genes and the gene order in the common bean were highly conserved and exhibited extensive synteny with cowpea. However, none of the orthologs for Phvul.002G270800 in the syntenic block were identified (Figure 7A). To identify the orthologous gene of Phvul.002G270800 (*PvGUX1_1*), the Phvul.002G270800 protein was used to conduct a BLASTP search against cowpea, soybean, pea, and common bean. Specifically, we identified another common bean protein, Phvul.009G148800 (*PvGUX1_2*), which shared a strong sequence homolog to *PvGUX1_1*. *PvGUX1_2* encoded 636 amino acids, whereas *PvGUX1_1* encoded 221 amino acids. The best hit of *PvGUX1_1* in cowpea, Vigun03g064600, encoded 629 amino acids, which exhibited larger sequence differences than *PvGUX1_1*. To verify the relationship between *PvGUX1_1*, *PvGUX1_2*, and GUX1, we performed a phylogenetic analysis of *PvGUX1_1*, *PvGUX1_2*, and other orthologous genes. *PvGUX1_1* and *PvGUX1_2* were placed in two different sub-branches (Figure 7B). Although the corresponding genes, Glyma08g15640 and Vigun03g064600, in the synteny region were clustered with *PvGUX1_1* in one sub-branch, there was large sequence variation between *PvGUX1_1* and other orthologs. Collectively, these data demonstrated that *PvGUX1_1* and *PvGUX1_2* diverged at an early stage in legume evolution, which may have resulted in gene diversification.

### 3.7. Gene Expression Patterns of PvGUX1_1

The formation of pod sutures is an important agronomic trait. To better reveal the genetic regulation of pod sutures, we conducted a qRT-PCR analysis of *PvGUX1_1* at the initiation of pod elongation (T1, no suture), pod development (T2, no suture), and pod maturation (T3, sutures were present in sutured pods) for sutured (R05) and non-sutured pods (R02) (Figure 7D). The qRT-PCR results indicated that the expression levels of *PvGUX1_1* were significantly higher at the T1 and T2 stages in non-sutured pods compared with the sutured pods (Figure 7E). Furthermore, the expression level of *PvGUX1_1* decreased following the development of pods in non-sutured pods (Figure 7C).

## 4. Discussion

Understanding the genetic mechanism of suture string development will facilitate the study of the domestication and plant breeding of snap beans. Here, we identified a strong signal on Chr02 that determined the formation of pod stringlessness based on large-scale resequencing. Within these putative genes in candidate regions, *PvGUX1_1* was the best candidate gene due to its function and polymorphisms, which was consistent with dominant inheritance.

### 4.1. GWAS Analysis for Pod Stringlessness

As the common bean is a selfing species, effective recombination and heterozygosis in the common bean are significantly reduced, which results in the generation of a large LD and slow LD decay. Generally, LD decay is slower in selfing species than in outcrossing species because of the loss of recombination, which potentially leads to be homozygosity [35]. The nature of homozygosity allows the common bean to design GWAS. In particular, once a genotype is sequenced, the phenotype can be investigated in different environments. Moreover, the extensive genetic diversity is advantageous for GWAS analysis in the common bean [36].

### 4.2. Pod Stringlessness Is Controlled by a Major Locus

The inheritance of pod stringlessness is complex due to genotypic and environmental factors [37]. Since the stringless trait was first observed, various inheritance models for the stringless trait in the common bean have been proposed. A model of two genes (S for dominant, T epistatic to S) was proposed to explain responsibility for the stringless trait [38]. However, other studies have revealed that the stringless trait is under the control of a single dominant locus (*St*), which is mapped on chromosome 2 [7,8,39]. Moreover, there have also been reports that the trait does not fit the ratio of one or more loci, and thus is a quantitative trait [2]. In order to verify the inheritance pattern, qualitative traits and quantitative traits were both used for the GWAS analysis. Interestingly, we obtained similar results from the two models. The only strong signal from both models was identified on Chr02, which was in agreement with previous findings, and showed that the formation of suture strings was controlled by a major locus.

The formation of suture strings is controlled by a single locus, while the level (short versus long) of the string might be more complex. This characteristic was also observed in pod shattering. Previous reports showed that at least two additional loci were likely relevant to the level and mode of pod shattering [9]. In our study, in addition to Chr02, an SNP located at Chr11 was also associated with stringlessness (Supplementary Figure S3). The SNP was about 0.13 Mb from the NAC transcription factor gene *PvCUC2* (*Phvul.011G160400*, Chr11:45614432_45616861). In order to identify more loci, we conducted GWAS only on stringed snap beans for ST1-2019. Strong association signals were identified on chromosome 7 (Supplementary Figure S4). These loci may be responsible for the level of suture strings, along with the St gene. This finding supported the hypothesis that a major factor influenced the string formation trait, while other genes led to incomplete strings [40].

### 4.3. Candidate Gene for Stringlessness in Snap Beans

A total of 23 genes within the LD block surrounding the high association signal were identified. Among them, several of the genes are orthologous genes involved in cell-wall biosynthesis, pod shattering, and fiber formation. *Phvul.002G270800* is the orthologous gene of *AtGUX1* (*AT3G18660*). In Arabidopsis, AtGUX1 belongs to Glycosyltransferase Family 8, which participates in the synthesis of plant cell walls [41]. *AtGUX1* is responsible for the decoration of xylan, an important component of the secondary cell wall [42]. Silencing *AtGUX1* led to a decrease in glucuronoxylan content and microsomal xylan in the cell wall [43]. Unlike the *AtGUX1* gene in Arabidopsis, which has only one copy, two copies are found in the common bean. This result indicated that *GUX1* underwent a broad expansion in the common bean as compared to Arabidopsis. The phylogenetic analysis based on protein sequences classified the two copies into two clades. The differences might be associated with biological function differentiation. Furthermore, more than 400 aa residues located at the N-terminal were lost in *PvGUX1_1* compared with other GUX1 homologous proteins, suggesting its specific function. Taken together, these findings indicate that the functions of two *PvGUX1* genes have diversified during evolution. This phenomenon has been found in Arabidopsis, in which the members of the GUX family have different functions and distinct expression patterns [41].

In our study, a 2 bp deletion was found in the exon region of *PvGUX1,* causing a premature stop. The 2 bp deletion was significantly associated with pod stringlessness. Although *Phvul.002G271600* also possessed 2 bp deletion in the exon and caused a premature stop codon, it was weakly related to pod stringlessness. Therefore, we propose *Phvul.002G270800* as the best candidate gene for the St locus. In addition to *Phvul.002G270800,* another gene of interest was *Phvul.002G271000,* the orthologous gene to *AtIND. AtIND,* as a b-HLH transcription factor, plays a crucial role in pod shattering in Arabidopsis [44,45,46]. However, due to the lack of mutation in *PvIND* (*Phvul.002G270800*), a previous study reported that it was not the *St* gene controlling the suture strings [8]. Although the present study identified three nonsynonymous SNPs in the exon region of *PvIND*, these SNPs only showed a weak association with the suture strings. Therefore, *PvIND* may not be directly involved in suture development. Other interesting genes included NAC transcription factor *Phvul.002G271700* (*PvVNI1*) and MYB transcription factor *Phvul.002G269900* (*PvMAMYB*). Many studies have suggested that an NAC transcription factor is correlated with pod shattering and secondary cell wall development [47,48,49]. In particular, the role of the NAC transcription factor SHA1-5 in regulating pod shattering in soybean has been elucidated in detail [30]. Likewise, several MYB transcription factors, such as *MYB26* [50], *MYB46* [51,52], and *MYB63* [53], are involved in lignin biosynthesis and secondary cell-wall formation in many species [54]. Therefore, the functions of *PvVNI1* and *PvMAMYB* need to be further studied in future research to determine whether they are related to suture string development or pod shattering.

### 4.4. Pod Stringlessness in Other Legumes

The loss or presence of suture strings is not an important factor for many legumes whose dry seeds are consumed. In legumes, reports on the stringless trait are currently only found for the common bean and pea. In pea, pod stringlessness arose from spontaneous mutation [55]. The recessive gene (*sin-2*) is regarded as the key gene responsible for the stringless trait in pea [37,56]. In contrast, the stringless trait in the common bean is governed by the dominant *St* gene. In the synteny block, the orthologs of *GUX1* were not detected in pea, indicating that the genetic mechanism of the stringlessness of the two legumes may be different.

Although the same regulation gene may not be shared by both the common bean and pea, there are many parallels, including seed dormancy, growth habit, and earliness, between the common bean and pea that have occurred in the process of crop domestication [57]. The identification of the *St* gene in the common bean would accelerate the mining of *sin-2* and improve the understanding of the genetics of domestication under parallel selection in the future.

## 5. Conclusions

In the present study, the suture strings of 138 snap bean accessions were investigated across two years. The whole-genome resequencing of these accessions generated 3.66 million single-nucleotide polymorphisms (SNPs). The analysis of population structure indicated that a high degree of intercrossing between Andean and Middle American gene pools occurred within Chinese snap beans. GWAS identified a strong candidate region for pod stringlessness. Within the region, a total of 23 putative genes were annotated. The 2 bp deletion in the exon of *PvGUX1_1* resulted in a truncated protein in stringed pods and was significantly associated with pod stringlessness. Moreover, the gene expression of *PvGUX1_1* was also significantly different in sutured pods and non-sutured pods. Taken together, the results suggest that *PvGUX1_1* is the best candidate gene for pod stringlessness. This study provides useful insights into the molecular mechanism of suture string formation and valuable information for snap bean improvement.

## Figures and Tables

**Table 1:** Putative genes in the 266.19 kb of candidate region.

Gene	Position	Homologs of Arabidopsis	Functional Annotation
*Phvul.002G269700*	Chr02:44022772_44024106		Unknown
*Phvul.002G269800*	Chr02:44033313_44034850	AT4G08250	GRAS family transcription factor
*Phvul.002G269900*	Chr02:44036772_44037686	AT5G45420	MYB transcription factor
*Phvul.002G270000*	Chr02:44037878_44041667	AT3G18750	WNK family of protein kinases
*Phvul.002G270100*	Chr02:44042454_44059878	AT3G18730	Involved in cell division control and plant morphogenesis
*Phvul.002G270200*	Chr02:44066265_44067018	AT5G64667	Involved in floral organ abscission
*Phvul.002G270300*	Chr02:44080456_44082336	AT5G64660	CYS, MET, PRO, and GLY protein
*Phvul.002G270400*	Chr02:44125511_44131328	AT5G24320	Transducin/WD40 repeat-like superfamily protein
*Phvul.002G270500*	Chr02:44133980_44137288	AT3G18680	UMP Kinase
*Phvul.002G270600*	Chr02:44139105_44139713	AT3G18690	Involved in mediating responses to pathogens
*Phvul.002G270700*	Chr02:44142989_44144630	AT4G14620	Unknown
*Phvul.002G270800*	Chr02:44150261_44150926	AT3G18660	Encodes a glucuronyltransferase responsible for the addition of GlcA residues onto xylan and for secondary wall deposition
*Phvul.002G270900*	Chr02:44155318_44157803	AT5G09760	Plant invertase/pectin methylesterase inhibitor
*Phvul.002G271000*	Chr02:44186987_44188326	AT4G00120	IND(basic helix-loop-helix (bHLH) DNA-binding superfamily protein)
*Phvul.002G271100*	Chr02:44199222_44205529	AT1G48880	Encodes a member of the TBL
*Phvul.002G271200*	Chr02:44205946_44210215	AT5G64630	Involved in organization of the shoot and root apical meristems
*Phvul.002G271300*	Chr02:44216969_44222195		Unknown
*Phvul.002G271400*	Chr02:44224184_44228271	AT1G08490	Chloroplastic NifS-like protein
*Phvul.002G271500*	Chr02:44229799_44246330	AT5G64070	Encodes a phosphatidylinositol 4-OH kinase
*Phvul.002G271600*	Chr02:44232557_44233756		Unknown
*Phvul.002G271700*	Chr02:44247536_44251023	AT5G09330	NAC domain containing protein
*Phvul.002G271800*	Chr02:44251436_44254178	AT2G13690	PRLI-interacting factor
*Phvul.002G271900*	Chr02:44257054_44258132		Unknown

**Table 2:** Functional annotation information of candidate genes.

Gene	Variant Type	Base Change	Amino Change
*Phvul.002G269700*	Nonsynonymous	A491G	E164G
*Phvul.002G270100*	Nonsynonymous	T1617A	D539E
	Nonsynonymous	A1899T	R633S
	Nonsynonymous	G2092A	D698N
	Nonsynonymous	C3050T	S1017L
*Phvul.002G270300*	Nonsynonymous	C257T	S86L
	Nonsynonymous	T382C	F128L
*Phvul.002G270400*	Nonsynonymous	G563A	R188K
	Nonsynonymous	C833A	T278N
*Phvul.002G270800*	Nonsynonymous	A92G	D31G
	Frameshift deletion	AT163_	
*Phvul.002G270900*	Nonsynonymous	G1374C	E458D
*Phvul.002G271000*	Nonsynonymous	T32C	V11A
	Nonsynonymous	C604T	P202S
	Nonsynonymous	C737T	T246M
*Phvul.002G271100*	Nonsynonymous	A257G	N86S
*Phvul.002G271200*	Nonsynonymous	G870A	M290I
*Phvul.002G271300*	Nonsynonymous	G241T	G81C
	Nonsynonymous	A638C	E213A
	Nonsynonymous	A781G	T261A
	Nonsynonymous	T1945G	S649A
	Nonsynonymous	A2008G	N670D
*Phvul.002G271400*	Nonsynonymous	G234A	M78I
	Nonsynonymous	A335C	K112T
	Nonsynonymous	C902T	A301V
	Nonsynonymous	A1099G	T367A
*Phvul.002G271600*	Nonsynonymous	T17C	L6S
	Nonsynonymous	A24C	L8F
	Frameshift deletion	GT57_	
*Phvul.002G271700*	Nonsynonymous	A77G	N26S
	Nonsynonymous	C78G	N26K
	Nonsynonymous	T100G	F34V
	Nonsynonymous	T154C	S52P
	Nonsynonymous	G162C	K54N
	Nonsynonymous	A359T	K120I
	Nonsynonymous	T476C	V159A
	Nonsynonymous	A785C	D262A
*Phvul.002G271800*	Nonsynonymous	A1346C	Y449S
	Nonsynonymous	A1138C	K380Q
	Nonsynonymous	A772C	N258H
	Nonsynonymous	T563A	F188Y
	Nonsynonymous	A385G	N129D
	Nonsynonymous	C328A	L110M
*Phvul.002G271900*	Nonsynonymous	T5G	F2C
	Nonsynonymous	T377C	V126A

## Data Availability

The raw resequencing data used in the study have been deposited in Genome Sequence Archive [58] in the BIG Data Center, Beijing Institute of Genomics (BIG), Chinese Academy of Sciences, under accession number PRJCA008878 and are publicly accessible at http://bigd.big.ac.cn, accessed on 1 April 2022.

## Supplementary Materials

The following are available online at https://www.mdpi.com/article/10.3390/biology11040611/s1, Figure S1: Decay of linkage disequilibrium (LD) in the snap bean genome; Figure S2: Sequence alignment of Phvul.002G270800 in four different accessions. R02 and R12 are stringless; R05 and R17 have suture strings; Figure S3: The Manhattan plots of ST1-2019 on chromosome 11. The red dashed line represents the threshold; Figure S4: The Manhattan plots of GWAS for stringy snap bean on ST1-2019; Table S1: List of common bean germplasm used in this study including ID, name, type of accession, variation of 2bp and soure of data. + indicates exist of 2bp; − for 2bp deletion; u for unknown. IVF_CAAS for Institute of Vegetables and Flowers, Chinese Academy of Agricultural Sciences; Table S2: Primer sequence used in qRT-PCR; Table S3: SNP distribution on chromosomes; Table S4: LD decay on each chromosome; Table S5: Significantly association signal for four traits by GWAS analysis.
