# Supplementary material for: Genome-Wide Association Study Reveals That PvGUX1_1 Is Associated with Pod Stringlessness in Snap Bean (Phaseolus vulgaris L.)
Source: Biology (Basel). 2022 Apr 18;11(4):611. doi: 10.3390/biology11040611 (PMC9024788; doi:10.3390/biology11040611)
Supplement: Supplementary file 1 [file biology-11-00611-s001.zip › biology-1638606-supplementary.pdf]

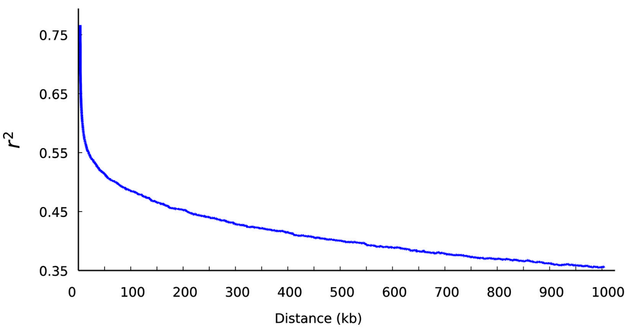

Figure S1. Decay of linkage disequilibrium (LD) in the snap bean genome.

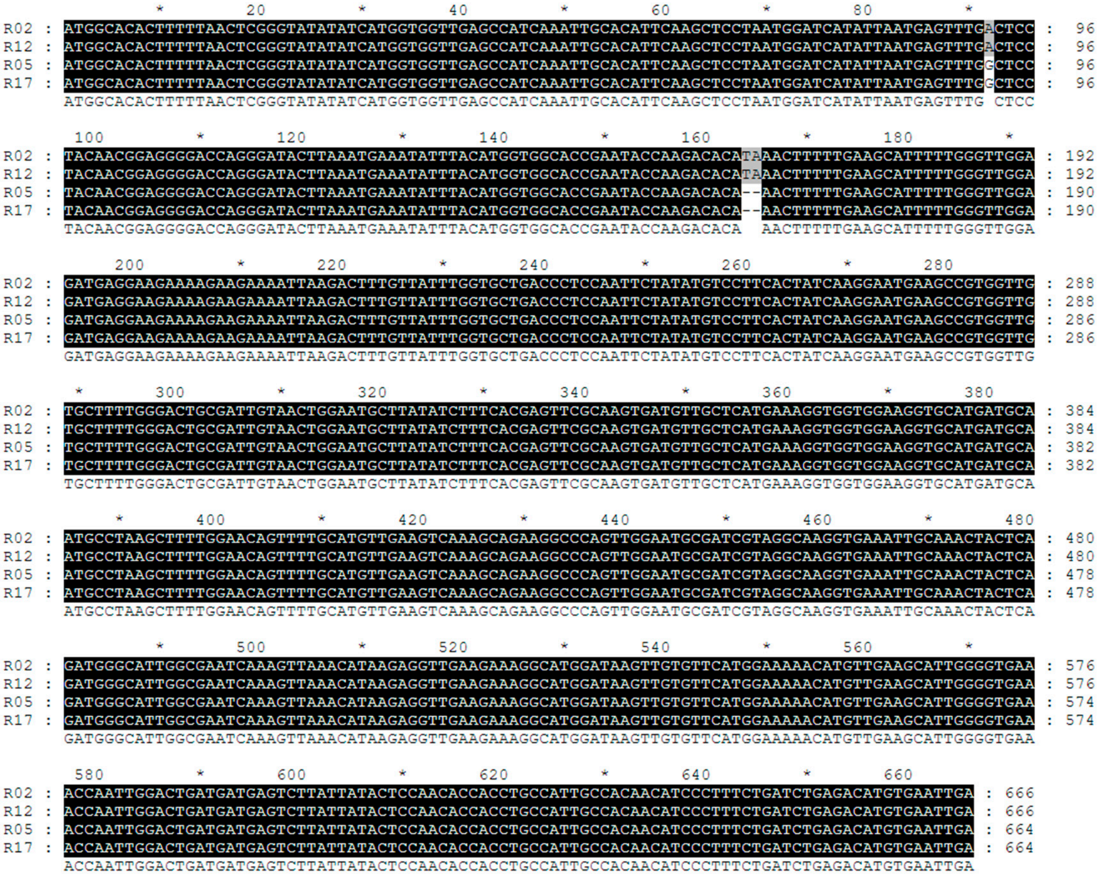

Figure S2. Sequence alignment of Phvul.002G270800 in four different accessions. R02 and R12 are stringless; R05 and R17 have suture strings.

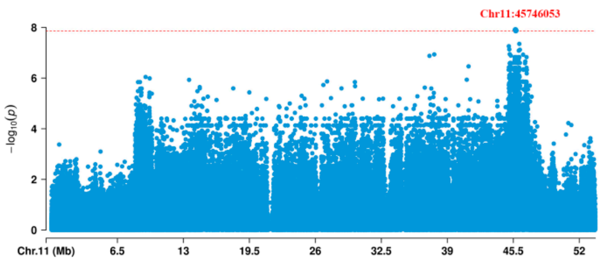

Figure S3. The Manhattan plots of ST1-2019 on chromosome 11. The red dashed line represents the threshold.

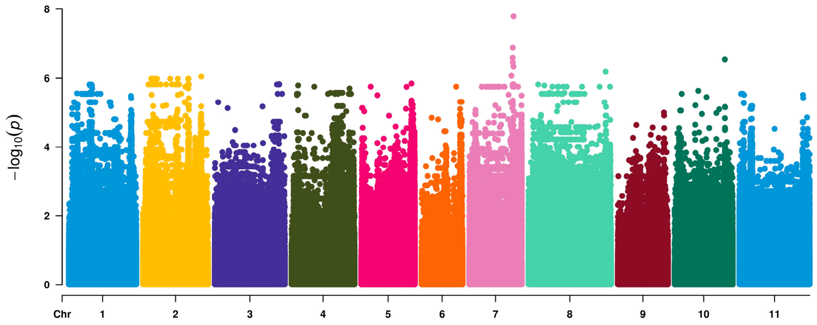

Figure S4. The Manhattan plots of GWAS for stringy snap bean on ST1-2019.

Table S1. List of common bean germplasm used in this study including ID, name, type of accession, variation of 2bp and source of data. + indicates exist of 2bp; - for 2bp deletion; u for unknown. IVF_CAAS for Institute of Vegetables and Flowers, Chinese Academy of Agricultural Sciences.

ID	Name	Genepool	2-bp	Source	ST1_2019	ST2_2019	ST1_2020	ST2_2020
R01	8030	Mesoamerican	+	IVF_CAAS	0	0	0	0
R02	15B5	Mesoamerican	+	IVF_CAAS	0	0	0	0
R03	17B1-1	Mesoamerican	-	IVF_CAAS	1	1	1	1
R04	14-2	Mesoamerican	-	IVF_CAAS	1	1	0.54	1
R05	09B2	Mesoamerican	-	IVF_CAAS	1	1	1	1
R06	96B44-1	Mesoamerican	-	IVF_CAAS	1	1	1	1
R07	17B2	Mesoamerican	-	IVF_CAAS	1	1	1	1
R08	09B5	Mesoamerican	-	IVF_CAAS	1	1	1	1
R09	15B8	Mesoamerican	-	IVF_CAAS	1	1	1	1
R10	16B1	Mesoamerican	+	IVF_CAAS	0	0	0	0
R11	17B4	Mesoamerican	-	IVF_CAAS	1	1	1	1
R12	10B7	Mesoamerican	+	IVF_CAAS	0	0	0	0
R13	10B4	Mesoamerican	+	IVF_CAAS	0.36	1	0	0
R14	15-28	Mesoamerican	+	IVF_CAAS	0	0	0	0
R15	17B3-1	Mesoamerican	-	IVF_CAAS	1	1	1	1
R16	18B4	Mesoamerican	-	IVF_CAAS	1	1	1	1
R17	16B3	Mesoamerican	-	IVF_CAAS	1	1	1	1
R18	15-68	Mesoamerican	-	IVF_CAAS	1	1	1	1
R19	15-69	Mesoamerican	+	IVF_CAAS	0	0	0	0
R20	15-73	Mesoamerican	+	IVF_CAAS	0	0	0	0
R21	82-3	Mesoamerican	+	IVF_CAAS	0	0	0	0
R22	82-62	Andean	+	IVF_CAAS	0	0	0	0
R23	12B3	Mesoamerican	+	IVF_CAAS	0	0	0	0
R25	15-20	Mesoamerican	+	IVF_CAAS	0	0	0	0
R26	15-22-1	Mesoamerican	+	IVF_CAAS	0	0	0	0
R27	15-30-1	Mesoamerican	+	IVF_CAAS	0.17	1	0	0
R28	15-32-2	Mesoamerican	+	IVF_CAAS	0.27	1	0	0
R29	15-39-1	Mesoamerican	+	IVF_CAAS	0	0	0	0
R30	15-74-2	Mesoamerican	+	IVF_CAAS	0	0	0	0
R31	17B12	Mesoamerican	-	IVF_CAAS	0.68	1	1	1
R32	17B8	Mesoamerican	-	IVF_CAAS	1	1	1	1
R33	16B2	Andean	+	IVF_CAAS	0	0	0	0
R34	98-5	Mesoamerican	+	IVF_CAAS	0	0	0	0
B35	C18	Mesoamerican	-	IVF_CAAS	1	1	1	1
B36	C21	Mesoamerican	+	IVF_CAAS	1	1	1	1
B37	Shuang2	Mesoamerican	-	IVF_CAAS	1	1	1	1
B38	Shuang3	Mesoamerican	-	IVF_CAAS	1	1	1	1
B39	S1	Mesoamerican	-	IVF_CAAS	1	1	1	1
B40	H1	Andean	+	IVF_CAAS	0	0	0	0
B41	H3	Andean	+	IVF_CAAS	0	0	0	0
B42	H4	Andean	+	IVF_CAAS	0	0	0	0
B43	C1	Mesoamerican	-	IVF_CAAS	0.82	1	0.21	1
B44	C2	Andean	+	IVF_CAAS	0	0	0	0
B45	C4	Mesoamerican	-	IVF_CAAS	1	1	1	1
B46	C5	Mesoamerican	-	IVF_CAAS	1	1	1	1
B47	C8	Mesoamerican	-	IVF_CAAS	1	1	0.25	1
B48	C9	Andean	+	IVF_CAAS	0.64	1	0.3	1
B49	C10	Mesoamerican	-	IVF_CAAS	1	1	1	1
B50	C11	Mesoamerican	-	IVF_CAAS	1	1	1	1
B51	C19	Mesoamerican	-	IVF_CAAS	1	1	1	1

B52	C20	Andean	+	IVF_CAAS	0	0	0	0
B53	C22	Andean	+	IVF_CAAS	0	0	0	0
B54	C23	Mesoamerican	-	IVF_CAAS	1	1	1	1
B55	C24	Andean	+	IVF_CAAS	0	0	0	0
B56	C26	Mesoamerican	-	IVF_CAAS	1	1	1	1
B57	C29	Andean	+	IVF_CAAS	0	0	0	0
B58	K1	Mesoamerican	+	IVF_CAAS	0	0	0	0
B59	K6	Mesoamerican	-	IVF_CAAS	1	1	0.24	1
B60	D6	Mesoamerican	-	IVF_CAAS	1	1	1	1
B61	T8	Andean	+	IVF_CAAS	0	0	0	0
B62	T20	Andean	+	IVF_CAAS	0	0	0	0
B63	9100	Mesoamerican	-	IVF_CAAS	1	1	1	1
B64	9128 7A179	Mesoamerican	-	IVF_CAAS	1	1	1	1
B65	9240 7A1875	Mesoamerican	-	IVF_CAAS	1	1	1	1
B66	9251 7A1955	Mesoamerican	-	IVF_CAAS	1	1	0.28	1
B67	9252 7A1957	Mesoamerican	-	IVF_CAAS	1	1	1	1
B68	9270 7A237	Mesoamerican	-	IVF_CAAS	1	1	1	1
B69	9270 7A237	Mesoamerican	-	IVF_CAAS	1	1	1	1
B70	9272 7A240	Mesoamerican	-	IVF_CAAS	1	1	1	1
B71	7A431	Mesoamerican	-	IVF_CAAS	1	1	1	1
B72	7A140	Andean	-	IVF_CAAS	1	1	1	1
B73	7A1834	Mesoamerican	-	IVF_CAAS	1	1	0.31	1
B74	7A1826	Mesoamerican	-	IVF_CAAS	1	1	1	1
B75	7A1423	Andean	+	IVF_CAAS	0	0	0	0
B76	7A1086	Mesoamerican	-	IVF_CAAS	1	1	0.34	1
B77	7A1119	Andean	+	IVF_CAAS	0	0	0	0
B78	9081	Mesoamerican	-	IVF_CAAS	1	1	1	1
B79	9244 7A1916	Mesoamerican	-	IVF_CAAS	1	1	1	1
B80	C13	Andean	+	IVF_CAAS	0	0	0	0
B81	C14	Mesoamerican	-	IVF_CAAS	1	1	1	1
B82	9055	Mesoamerican	-	IVF_CAAS	1	1	1	1
B83	C30	Andean	+	IVF_CAAS	0	0	0	0
B84	T5	Andean	+	IVF_CAAS	0	0	0	0
B85	T6ao	Andean	+	IVF_CAAS	0	0	0	0
B86	T6tu	Andean	+	IVF_CAAS	0	0	0	0
B87	C27	Andean	+	IVF_CAAS	0	0	0	0
B88	T7	Andean	+	IVF_CAAS	0	0	0	0
B89	7A372	Mesoamerican	-	IVF_CAAS	1	1	1	1
B90	C15	Andean	+	IVF_CAAS	0	0	0	0
B91	T6	Andean	+	IVF_CAAS	0	0	0	0
B92	K9	Mesoamerican	-	IVF_CAAS	1	1	0.72	1
B93	T23	Andean	+	IVF_CAAS	0	0	0	0
B94	T3	Andean	+	IVF_CAAS	0	0	0	0
B95	C7	Andean	+	IVF_CAAS	0	0	0	0
B96	C17	Andean	+	IVF_CAAS	0.08	1	0.69	1
B97	80-107	Mesoamerican	-	IVF_CAAS	1	1	1	1
B98	C3	Andean	+	IVF_CAAS	0	0	0.54	1
B99	C12	Andean	+	IVF_CAAS	0	0	0	0
B100	C16	Andean	+	IVF_CAAS	0	0	0	0
B101	C6	Andean	+	IVF_CAAS	0	0	0	0
B102	T17	Andean	+	IVF_CAAS	0	0	0	0
B103	Shuang1	Mesoamerican	-	IVF_CAAS	1	1	1	1
B104	T27	Mesoamerican	-	IVF_CAAS	1	1	0.64	1
B105	H2	Andean	+	IVF_CAAS	0	0	0	0
B106	2504	Mesoamerican	+	IVF_CAAS	0.77	1	0.69	1
B107	V07A0978	Mesoamerican	-	IVF_CAAS	1	1	1	1
B108	H7	Andean	+	IVF_CAAS	0	0	0	0
B109	H6	Andean	+	IVF_CAAS	0	0	0	0

B110	H5	Andean	+	IVF_CAAS	0	0	0	0
B111	K2	Mesoamerican	-	IVF_CAAS	1	1	1	1
B112	7A1795	Mesoamerican	-	IVF_CAAS	1	1	1	1
B113	T22	Andean	+	IVF_CAAS	0.85	1	1	1
B114	H8	Andean	+	IVF_CAAS	0	0	0	0
B115	7A394	Mesoamerican	-	IVF_CAAS	1	1	1	1
B116	C25	Andean	+	IVF_CAAS	0	0	0	0
B117	2404	Mesoamerican	+	IVF_CAAS	0	0	0	0
B118	86-2	Mesoamerican	+	IVF_CAAS	0	0	0	0
B119	T32	Andean	+	IVF_CAAS	1	1	1	1
B120	80-143	Mesoamerican	-	IVF_CAAS	1	1	1	1
B121	C28	Andean	+	IVF_CAAS	0	0	0	0
B122	19B1	Mesoamerican	-	IVF_CAAS	1	1	1	1
B123	19B2	Mesoamerican	+	IVF_CAAS	0	0	u	u
B124	19B4	Mesoamerican	-	IVF_CAAS	1	1	1	1
B125	19B5	Mesoamerican	-	IVF_CAAS	1	1	1	1
B126	19B6	Andean	+	IVF_CAAS	0.04	1	0	0
B127	19B7-1	Andean	+	IVF_CAAS	0	0	0	0
B128	19B7-2	Mesoamerican	-	IVF_CAAS	1	1	1	1
B129	19B8	Mesoamerican	-	IVF_CAAS	1	1	1	1
B130	19B9	Mesoamerican	-	IVF_CAAS	1	1	u	u
B131	19B10	Mesoamerican	-	IVF_CAAS	1	1	0.71	1
B132	19B11	Mesoamerican	-	IVF_CAAS	1	1	1	1
B133	19B12	Mesoamerican	-	IVF_CAAS	1	1	0.83	1
B134	19B13	Mesoamerican	+	IVF_CAAS	0	0	0	0
B135	19B14	Mesoamerican	-	IVF_CAAS	1	1	1	1
B136	19B15	Mesoamerican	-	IVF_CAAS	1	1	0.68	1
B137	B20B1	Mesoamerican	+	IVF_CAAS	0	0	0	0
B138	B20B2	Mesoamerican	-	IVF_CAAS	1	1	1	1
B139	B20B3	Andean	+	IVF_CAAS	0	0	0	0

Table S2. Primer sequence used in qRT-PCR.

Primer name	Primer sequence(5'-3')
PvGUX1_1	GGTTGAAGAAAGGCATGGATAAG
	GCAGGTGGTGTTGGAGTATAA
Actin	GAAGTTCTCT TCCAACCATCC
	TTCCTTGCTCATT CTGTCCG

Table S3. SNP distribution on chromosomes.

Chromosome	Length of Chromosome(bp)	SNP number	SNP density (No./kb)
Chr01	51433939	360596	7.01
Chr02	49670989	411294	8.28
Chr03	53438756	384632	7.2
Chr04	48048378	339438	7.06
Chr05	40923498	278286	6.8
Chr06	31236378	238452	7.63
Chr07	40041001	269564	6.73

Chr08	63048260	376474	5.97
Chr09	38250102	310259	8.11
Chr10	44302882	290060	6.55
Chr11	53580169	330923	6.18

Table S4. LD decay on each chromosome.

Chromosome	LD decay
Chr01	976,100
Chr02	491,800
Chr03	478,700
Chr04	350,300
Chr05	284,100
Chr06	904,100
Chr07	546,000
Chr08	473,300
Chr09	876,300
Chr10	184,100
Chr11	963,000

Table S5. Significantly association signal for four traits by GWAS analysis.

Traits	SNP	Chromosome	Position	P.value	Rsquare.of.Model.without.SNP	Rsquare.of.Model.with.SNP
ST1-2019	SNP709759	Chr02	44026689	8.34E-11	0.66408708	0.809364444
ST1-2019	SNP710876	Chr02	44194695	4.22E-10	0.66408708	0.796539037
ST1-2019	SNP711816	Chr02	44328013	6.69E-10	0.66408708	0.792947758
ST1-2019	SNP711895	Chr02	44336662	7.34E-10	0.66408708	0.792232159
ST1-2019	SNP709536	Chr02	44002717	7.36E-10	0.66408708	0.79221138
ST1-2019	SNP711217	Chr02	44248269	8.14E-10	0.66408708	0.791438515
ST1-2019	SNP710933	Chr02	44206976	8.15E-10	0.66408708	0.791427149
ST1-2019	SNP711387	Chr02	44282675	8.25E-10	0.66408708	0.791333946
ST1-2019	SNP709538	Chr02	44002794	8.42E-10	0.66408708	0.791176773
ST1-2019	SNP709539	Chr02	44002817	8.42E-10	0.66408708	0.791176773

ST1-2019	SNP709540	Chr02	44002827	8.42E-10	0.66408708	0.791176773
ST1-2019	SNP709541	Chr02	44002840	8.42E-10	0.66408708	0.791176773
ST1-2019	SNP711694	Chr02	44321072	8.42E-10	0.66408708	0.791176773
ST1-2019	SNP712010	Chr02	44370231	1.46E-09	0.66408708	0.786975435
ST1-2019	SNP711219	Chr02	44248320	1.69E-09	0.66408708	0.785822964
ST1-2019	SNP711220	Chr02	44248346	1.69E-09	0.66408708	0.785822964
ST1-2019	SNP711221	Chr02	44248367	1.69E-09	0.66408708	0.785822964
ST1-2019	SNP711222	Chr02	44248372	1.69E-09	0.66408708	0.785822964
ST1-2019	SNP711218	Chr02	44248299	2.29E-09	0.66408708	0.783553738
ST1-2019	SNP711944	Chr02	44353434	2.30E-09	0.66408708	0.783510915
ST1-2019	SNP710706	Chr02	44169322	2.34E-09	0.66408708	0.783360845
ST1-2019	SNP712825	Chr02	44491321	2.82E-09	0.66408708	0.781978564
ST1-2019	SNP709797	Chr02	44031896	2.87E-09	0.66408708	0.781841832
ST1-2019	SNP709798	Chr02	44031898	2.87E-09	0.66408708	0.781841832
ST1-2019	SNP709799	Chr02	44031901	2.87E-09	0.66408708	0.781841832
ST1-2019	SNP711609	Chr02	44318734	3.39E-09	0.66408708	0.780580849
ST1-2019	SNP711611	Chr02	44318755	3.39E-09	0.66408708	0.780580849
ST1-2019	SNP709727	Chr02	44022619	4.06E-09	0.66408708	0.779234572
ST1-2019	SNP709537	Chr02	44002751	4.37E-09	0.66408708	0.778672472
ST1-2019	SNP711044	Chr02	44219414	4.96E-09	0.66408708	0.77772635
ST1-2019	SNP710014	Chr02	44075208	5.19E-09	0.66408708	0.777400934
ST1-2019	SNP709519	Chr02	44001188	5.27E-09	0.66408708	0.777287553

ST1-2019	SNP709591	Chr02	44007852	5.27E-09	0.66408708	0.777287553
ST1-2019	SNP709741	Chr02	44025318	5.27E-09	0.66408708	0.777287553
ST1-2019	SNP709764	Chr02	44027832	5.27E-09	0.66408708	0.777287553
ST1-2019	SNP709767	Chr02	44028010	5.27E-09	0.66408708	0.777287553
ST1-2019	SNP709893	Chr02	44052567	5.27E-09	0.66408708	0.777287553
ST1-2019	SNP709894	Chr02	44052573	5.27E-09	0.66408708	0.777287553
ST1-2019	SNP709922	Chr02	44062112	5.27E-09	0.66408708	0.777287553
ST1-2019	SNP710125	Chr02	44087774	5.27E-09	0.66408708	0.777287553
ST1-2019	SNP710126	Chr02	44087811	5.27E-09	0.66408708	0.777287553
ST1-2019	SNP710127	Chr02	44087825	5.27E-09	0.66408708	0.777287553
ST1-2019	SNP710312	Chr02	44116457	5.27E-09	0.66408708	0.777287553
ST1-2019	SNP710313	Chr02	44116481	5.27E-09	0.66408708	0.777287553
ST1-2019	SNP710314	Chr02	44116482	5.27E-09	0.66408708	0.777287553
ST1-2019	SNP710430	Chr02	44124153	5.27E-09	0.66408708	0.777287553
ST1-2019	SNP710435	Chr02	44124331	5.27E-09	0.66408708	0.777287553
ST1-2019	SNP710511	Chr02	44134874	5.27E-09	0.66408708	0.777287553
ST1-2019	SNP710512	Chr02	44134877	5.27E-09	0.66408708	0.777287553
ST1-2019	SNP710583	Chr02	44142506	5.27E-09	0.66408708	0.777287553
ST1-2019	SNP710584	Chr02	44142508	5.27E-09	0.66408708	0.777287553
ST1-2019	SNP710585	Chr02	44142510	5.27E-09	0.66408708	0.777287553
ST1-2019	SNP710913	Chr02	44198506	5.27E-09	0.66408708	0.777287553
ST1-2019	SNP710976	Chr02	44215281	5.27E-09	0.66408708	0.777287553

ST1-2019	SNP710977	Chr02	44215287	5.27E-09	0.66408708	0.777287553
ST1-2019	SNP711125	Chr02	44233252	5.27E-09	0.66408708	0.777287553
ST1-2019	SNP711137	Chr02	44236154	5.27E-09	0.66408708	0.777287553
ST1-2019	SNP711240	Chr02	44250218	5.27E-09	0.66408708	0.777287553
ST1-2019	SNP711445	Chr02	44284893	5.27E-09	0.66408708	0.777287553
ST1-2019	SNP711546	Chr02	44298694	5.27E-09	0.66408708	0.777287553
ST1-2019	SNP711547	Chr02	44298699	5.27E-09	0.66408708	0.777287553
ST1-2019	SNP711591	Chr02	44301978	5.27E-09	0.66408708	0.777287553
ST1-2019	SNP711697	Chr02	44321080	5.27E-09	0.66408708	0.777287553
ST1-2019	SNP711720	Chr02	44321354	5.27E-09	0.66408708	0.777287553
ST1-2019	SNP711721	Chr02	44321405	5.27E-09	0.66408708	0.777287553
ST1-2019	SNP711725	Chr02	44321473	5.27E-09	0.66408708	0.777287553
ST1-2019	SNP711873	Chr02	44332589	5.27E-09	0.66408708	0.777287553
ST1-2019	SNP712120	Chr02	44381836	5.27E-09	0.66408708	0.777287553
ST1-2019	SNP712181	Chr02	44387934	5.27E-09	0.66408708	0.777287553
ST1-2019	SNP712356	Chr02	44411445	5.27E-09	0.66408708	0.777287553
ST1-2019	SNP712580	Chr02	44454963	5.27E-09	0.66408708	0.777287553
ST1-2019	SNP712581	Chr02	44455001	5.27E-09	0.66408708	0.777287553
ST1-2019	SNP712638	Chr02	44460429	5.27E-09	0.66408708	0.777287553
ST1-2019	SNP712639	Chr02	44460430	5.27E-09	0.66408708	0.777287553
ST1-2019	SNP712651	Chr02	44460809	5.27E-09	0.66408708	0.777287553
ST1-2019	SNP712822	Chr02	44485863	5.27E-09	0.66408708	0.777287553

ST1-2019	SNP709758	Chr02	44026581	5.31E-09	0.66408708	0.777218034
ST1-2019	SNP711356	Chr02	44274949	5.61E-09	0.66408708	0.776810251
ST1-2019	SNP710484	Chr02	44131778	6.25E-09	0.66408708	0.776013424
ST1-2019	SNP710021	Chr02	44075340	6.29E-09	0.66408708	0.775960942
ST1-2019	SNP712733	Chr02	44467441	6.72E-09	0.66408708	0.775478009
ST1-2019	SNP710148	Chr02	44090056	7.38E-09	0.66408708	0.77478214
ST1-2019	SNP709736	Chr02	44024697	7.44E-09	0.66408708	0.774723688
ST1-2019	SNP709760	Chr02	44026855	7.81E-09	0.66408708	0.774361094
ST1-2019	SNP712767	Chr02	44478239	8.08E-09	0.66408708	0.774111105
ST1-2019	SNP712532	Chr02	44449855	8.40E-09	0.66408708	0.773818426
ST1-2019	SNP711839	Chr02	44328846	8.94E-09	0.66408708	0.773361029
ST1-2019	SNP709988	Chr02	44072693	9.19E-09	0.66408708	0.773157131
ST1-2019	SNP710875	Chr02	44194660	9.19E-09	0.66408708	0.773157131
ST1-2019	SNP712650	Chr02	44460803	9.27E-09	0.66408708	0.773091091
ST1-2019	SNP712835	Chr02	44492061	9.33E-09	0.66408708	0.773046164
ST1-2019	SNP710124	Chr02	44087741	9.42E-09	0.66408708	0.772971189
ST1-2019	SNP709575	Chr02	44006128	9.92E-09	0.66408708	0.772592204
ST1-2019	SNP709576	Chr02	44006140	9.92E-09	0.66408708	0.772592204
ST1-2019	SNP709593	Chr02	44008205	9.92E-09	0.66408708	0.772592204
ST1-2019	SNP709594	Chr02	44008228	9.92E-09	0.66408708	0.772592204
ST1-2019	SNP709650	Chr02	44015271	9.92E-09	0.66408708	0.772592204
ST1-2019	SNP709738	Chr02	44025236	9.92E-09	0.66408708	0.772592204

ST1-2019	SNP709739	Chr02	44025250	9.92E-09	0.66408708	0.772592204
ST1-2019	SNP709740	Chr02	44025268	9.92E-09	0.66408708	0.772592204
ST1-2019	SNP709744	Chr02	44025440	9.92E-09	0.66408708	0.772592204
ST1-2019	SNP709745	Chr02	44025441	9.92E-09	0.66408708	0.772592204
ST1-2019	SNP709746	Chr02	44025480	9.92E-09	0.66408708	0.772592204
ST1-2019	SNP709747	Chr02	44025483	9.92E-09	0.66408708	0.772592204
ST1-2019	SNP709748	Chr02	44025484	9.92E-09	0.66408708	0.772592204
ST1-2019	SNP709814	Chr02	44044002	9.92E-09	0.66408708	0.772592204
ST1-2019	SNP709827	Chr02	44045115	9.92E-09	0.66408708	0.772592204
ST1-2019	SNP709832	Chr02	44045457	9.92E-09	0.66408708	0.772592204
ST1-2019	SNP709897	Chr02	44053119	9.92E-09	0.66408708	0.772592204
ST1-2019	SNP709898	Chr02	44053121	9.92E-09	0.66408708	0.772592204
ST1-2019	SNP710089	Chr02	44082467	9.92E-09	0.66408708	0.772592204
ST1-2019	SNP710090	Chr02	44082487	9.92E-09	0.66408708	0.772592204
ST1-2019	SNP710128	Chr02	44087845	9.92E-09	0.66408708	0.772592204
ST1-2019	SNP710162	Chr02	44092347	9.92E-09	0.66408708	0.772592204
ST1-2019	SNP710163	Chr02	44092369	9.92E-09	0.66408708	0.772592204
ST1-2019	SNP710201	Chr02	44095520	9.92E-09	0.66408708	0.772592204
ST1-2019	SNP710453	Chr02	44128117	9.92E-09	0.66408708	0.772592204
ST1-2019	SNP710454	Chr02	44128763	9.92E-09	0.66408708	0.772592204
ST1-2019	SNP710468	Chr02	44130082	9.92E-09	0.66408708	0.772592204
ST1-2019	SNP710541	Chr02	44137836	9.92E-09	0.66408708	0.772592204

ST1-2019	SNP710576	Chr02	44142293	9.92E-09	0.66408708	0.772592204
ST1-2019	SNP710586	Chr02	44142549	9.92E-09	0.66408708	0.772592204
ST1-2019	SNP710587	Chr02	44142555	9.92E-09	0.66408708	0.772592204
ST1-2019	SNP710646	Chr02	44149334	9.92E-09	0.66408708	0.772592204
ST1-2019	SNP710743	Chr02	44175716	9.92E-09	0.66408708	0.772592204
ST1-2019	SNP710744	Chr02	44175729	9.92E-09	0.66408708	0.772592204
ST1-2019	SNP710941	Chr02	44209255	9.92E-09	0.66408708	0.772592204
ST1-2019	SNP711143	Chr02	44238327	9.92E-09	0.66408708	0.772592204
ST1-2019	SNP711152	Chr02	44240813	9.92E-09	0.66408708	0.772592204
ST1-2019	SNP711171	Chr02	44243617	9.92E-09	0.66408708	0.772592204
ST1-2019	SNP711203	Chr02	44247472	9.92E-09	0.66408708	0.772592204
ST1-2019	SNP711241	Chr02	44250344	9.92E-09	0.66408708	0.772592204
ST1-2019	SNP711358	Chr02	44276504	9.92E-09	0.66408708	0.772592204
ST1-2019	SNP711482	Chr02	44288382	9.92E-09	0.66408708	0.772592204
ST1-2019	SNP711532	Chr02	44296998	9.92E-09	0.66408708	0.772592204
ST1-2019	SNP711605	Chr02	44318480	9.92E-09	0.66408708	0.772592204
ST1-2019	SNP711606	Chr02	44318504	9.92E-09	0.66408708	0.772592204
ST1-2019	SNP711696	Chr02	44321079	9.92E-09	0.66408708	0.772592204
ST1-2019	SNP711770	Chr02	44325477	9.92E-09	0.66408708	0.772592204
ST1-2019	SNP711776	Chr02	44325919	9.92E-09	0.66408708	0.772592204
ST1-2019	SNP711968	Chr02	44361810	9.92E-09	0.66408708	0.772592204
ST1-2019	SNP712015	Chr02	44370987	9.92E-09	0.66408708	0.772592204

ST1-2019	SNP712016	Chr02	44371014	9.92E-09	0.66408708	0.772592204
ST1-2019	SNP712017	Chr02	44371018	9.92E-09	0.66408708	0.772592204
ST1-2019	SNP712047	Chr02	44373336	9.92E-09	0.66408708	0.772592204
ST1-2019	SNP712048	Chr02	44373344	9.92E-09	0.66408708	0.772592204
ST1-2019	SNP712121	Chr02	44381939	9.92E-09	0.66408708	0.772592204
ST1-2019	SNP712187	Chr02	44387969	9.92E-09	0.66408708	0.772592204
ST1-2019	SNP712188	Chr02	44387986	9.92E-09	0.66408708	0.772592204
ST1-2019	SNP712189	Chr02	44388002	9.92E-09	0.66408708	0.772592204
ST1-2019	SNP712229	Chr02	44389485	9.92E-09	0.66408708	0.772592204
ST1-2019	SNP712239	Chr02	44390426	9.92E-09	0.66408708	0.772592204
ST1-2019	SNP712292	Chr02	44398152	9.92E-09	0.66408708	0.772592204
ST1-2019	SNP712293	Chr02	44398154	9.92E-09	0.66408708	0.772592204
ST1-2019	SNP712294	Chr02	44398224	9.92E-09	0.66408708	0.772592204
ST1-2019	SNP712590	Chr02	44456407	9.92E-09	0.66408708	0.772592204
ST1-2019	SNP712637	Chr02	44460356	9.92E-09	0.66408708	0.772592204
ST1-2019	SNP712668	Chr02	44461729	9.92E-09	0.66408708	0.772592204
ST1-2019	SNP712669	Chr02	44461743	9.92E-09	0.66408708	0.772592204
ST1-2019	SNP712670	Chr02	44461757	9.92E-09	0.66408708	0.772592204
ST1-2019	SNP712741	Chr02	44469352	9.92E-09	0.66408708	0.772592204
ST1-2019	SNP712744	Chr02	44472356	9.92E-09	0.66408708	0.772592204
ST1-2019	SNP712757	Chr02	44476014	9.92E-09	0.66408708	0.772592204
ST1-2019	SNP712777	Chr02	44483735	9.92E-09	0.66408708	0.772592204

ST1-2019	SNP712842	Chr02	44492496	9.92E-09	0.66408708	0.772592204
ST1-2019	SNP711284	Chr02	44254302	1.02E-08	0.66408708	0.772397739
ST1-2019	SNP711533	Chr02	44297127	1.02E-08	0.66408708	0.772397739
ST1-2019	SNP711722	Chr02	44321421	1.02E-08	0.66408708	0.772397739
ST1-2019	SNP711723	Chr02	44321445	1.02E-08	0.66408708	0.772397739
ST1-2019	SNP711724	Chr02	44321446	1.02E-08	0.66408708	0.772397739
ST1-2019	SNP709265	Chr02	43982152	1.07E-08	0.66408708	0.772011841
ST1-2019	SNP712698	Chr02	44463539	1.09E-08	0.66408708	0.77190982
ST1-2019	SNP711610	Chr02	44318748	1.09E-08	0.66408708	0.771900621
ST1-2019	SNP712182	Chr02	44387942	1.10E-08	0.66408708	0.771844521
ST1-2019	SNP712183	Chr02	44387943	1.10E-08	0.66408708	0.771844521
ST1-2019	SNP710001	Chr02	44073270	1.12E-08	0.66408708	0.771732419
ST1-2019	SNP711929	Chr02	44348187	1.16E-08	0.66408708	0.771445978
ST1-2019	SNP708766	Chr02	43917710	1.19E-08	0.66408708	0.771230959
ST1-2019	SNP712111	Chr02	44378392	1.21E-08	0.66408708	0.771115718
ST1-2019	SNP710877	Chr02	44195229	1.26E-08	0.66408708	0.770816928
ST1-2019	SNP712603	Chr02	44458123	1.27E-08	0.66408708	0.770791425
ST1-2019	SNP712604	Chr02	44458132	1.27E-08	0.66408708	0.770791425
ST1-2019	SNP709644	Chr02	44014828	1.31E-08	0.66408708	0.770543644
ST1-2019	SNP709917	Chr02	44061478	1.31E-08	0.66408708	0.770543644
ST1-2019	SNP709986	Chr02	44072663	1.31E-08	0.66408708	0.770543644
ST1-2019	SNP710005	Chr02	44073583	1.31E-08	0.66408708	0.770543644

ST1-2019	SNP710176	Chr02	44093595	1.31E-08	0.66408708	0.770543644
ST1-2019	SNP710177	Chr02	44093598	1.31E-08	0.66408708	0.770543644
ST1-2019	SNP710211	Chr02	44096855	1.31E-08	0.66408708	0.770543644
ST1-2019	SNP710410	Chr02	44123138	1.31E-08	0.66408708	0.770543644
ST1-2019	SNP710637	Chr02	44149038	1.31E-08	0.66408708	0.770543644
ST1-2019	SNP711191	Chr02	44246953	1.31E-08	0.66408708	0.770543644
ST1-2019	SNP711239	Chr02	44250142	1.31E-08	0.66408708	0.770543644
ST1-2019	SNP711542	Chr02	44298186	1.31E-08	0.66408708	0.770543644
ST1-2019	SNP711543	Chr02	44298190	1.31E-08	0.66408708	0.770543644
ST1-2019	SNP711544	Chr02	44298191	1.31E-08	0.66408708	0.770543644
ST1-2019	SNP711545	Chr02	44298252	1.31E-08	0.66408708	0.770543644
ST1-2019	SNP712030	Chr02	44371636	1.31E-08	0.66408708	0.770543644
ST1-2019	SNP712050	Chr02	44373550	1.31E-08	0.66408708	0.770543644
ST1-2019	SNP712236	Chr02	44389680	1.31E-08	0.66408708	0.770543644
ST1-2019	SNP712388	Chr02	44413801	1.31E-08	0.66408708	0.770543644
ST1-2019	SNP712391	Chr02	44413984	1.31E-08	0.66408708	0.770543644
ST1-2019	SNP712746	Chr02	44473271	1.31E-08	0.66408708	0.770543644
ST1-2019	SNP712813	Chr02	44485663	1.31E-08	0.66408708	0.770543644
ST1-2019	SNP3507295	Chr11	45106268	1.36E-08	0.66408708	0.770257469
ST2-2019	SNP711217	Chr02	44248269	2.53E-09	0.600682726	0.721525683
ST2-2019	SNP711816	Chr02	44328013	3.64E-09	0.600682726	0.718421253
ST1-2020	SNP710871	Chr02	44194640	3.26E-09	0.542944548	0.691796257

ST2-2020	SNP710871	Chr02	44194640	2.42E-10	0.589833533	0.783118593
ST2-2020	SNP709736	Chr02	44024697	4.73E-09	0.589833533	0.747499937
